# Supplementary figures and images for: Deep learning-based forest fire detection using an improved SSD algorithm with CBAM
Source: PLoS One. 2025 Nov 18;20(11):e0333574. doi: 10.1371/journal.pone.0333574 (PMC12626326; doi:10.1371/journal.pone.0333574)

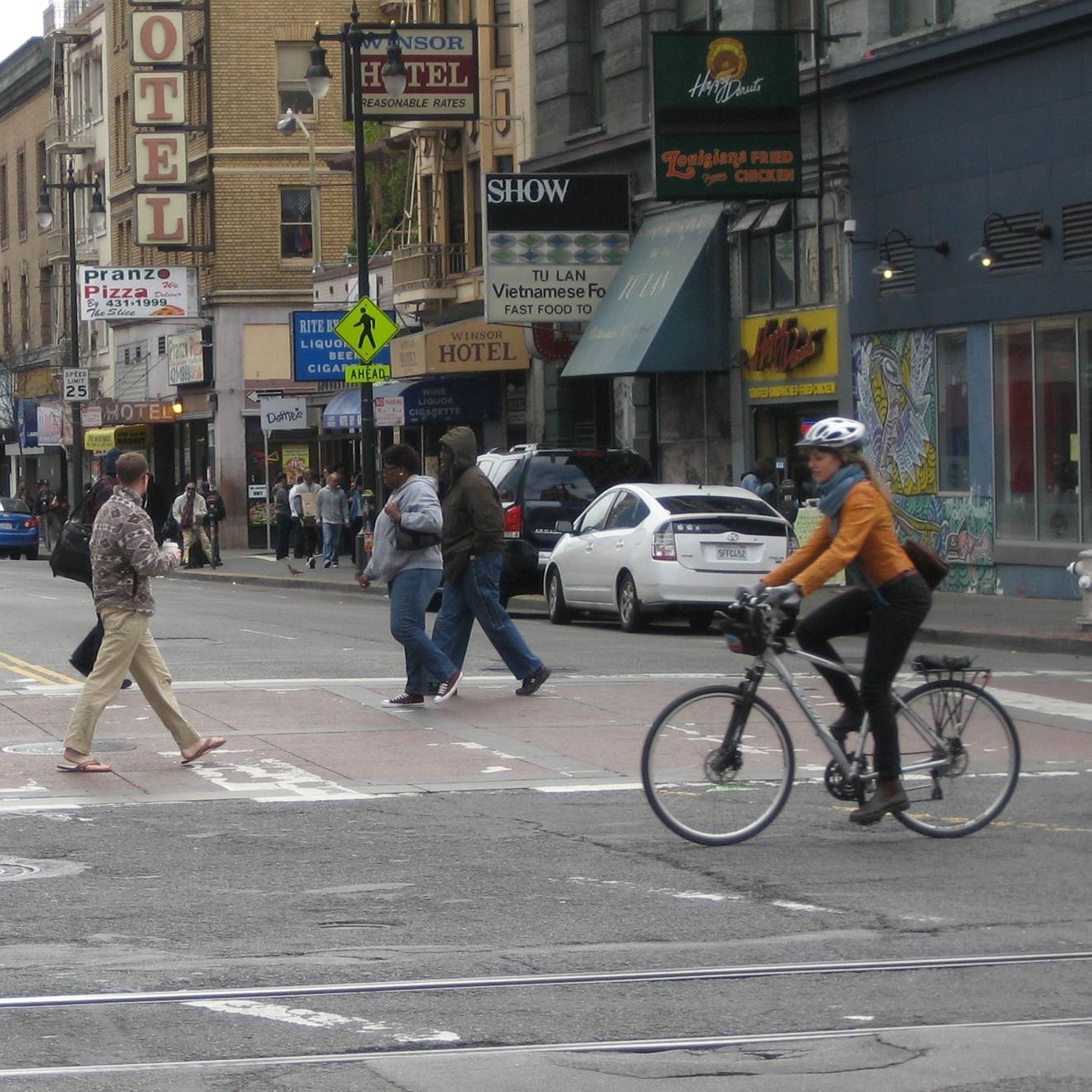

Supplement: S1 File — (ZIP) [file pone.0333574.s001.zip › improved-ssd-pytorch/ssd-pytorch-master/img/street.jpg]
