# Supplementary material for: Deep learning-based forest fire detection using an improved SSD algorithm with CBAM
Source: PLoS One. 2025 Nov 18;20(11):e0333574. doi: 10.1371/journal.pone.0333574 (PMC12626326; doi:10.1371/journal.pone.0333574)
Supplement: Attachment — Submitted filename: PONE-D-24-53870_review.docx [file pone.0333574.s002.docx]

**Research on Forest Fire Detection Based on Improved SSD Algorithm**

**General Comments**

The manuscript, "Research on Forest Fire Detection Based on Improved SSD Algorithm," presents an innovative approach to fire detection in forest environments by improving the Single Shot MultiBox Detector (SSD) algorithm with a Convolutional Block Attention Module (CBAM). The study is well-structured, methodologically sound, and provides empirical evidence supporting the improved performance of the proposed model. The results demonstrate that the enhanced SSD model achieves superior accuracy, recall, and robustness compared to traditional fire detection algorithms. This research has practical significance for real-time fire monitoring in forested regions, which is a critical area in environmental conservation and disaster prevention. The study addresses an important real-world problem: the need for automated, real-time, and high-accuracy forest fire detection.

This is a well-conducted study with significant contributions to the field of deep learning-based fire detection. The proposed CBAM-SSD model improves fire detection accuracy and robustness, making it a valuable advancement. However, before publication, the major revisions are strongly recommended.

**Specific Comments**

- **Title**
  - Consider specifying "deep learning-based detection" to make the title more informative. Suggested title revision: "Deep Learning-Based Forest Fire Detection Using an Improved SSD Algorithm with CBAM"
- **Abstract**
  - The phrase "high real-time and embeddability required for detection are achieved" is unclear and grammatically awkward.
  - The term "random data enhancement" is vague—it should specify data augmentation techniques used.
  - The purpose of "compensate for constraints in the data collection process" is unclear—what constraints?
  - "Constraint information" is unclear—does it mean feature extraction constraints?
  - "Channel (Channel) and space (Spatial)" is redundant—just say "channel and spatial features."
  - The phrase "serial structure is utilized" is vague—explain why this structure was chosen.
  - The phrase "traditional detection algorithm" is too vague—specify which algorithm (e.g., VGG-16-SSD).
  - The term "average accuracy AP" is redundant—just say "AP (Average Precision)".
  - The recall rate of 96.40%—is it for fire detection, smoke detection, or both?
  - mAP (97.55%)—better to clarify that it is the mean average precision across all categories.
  - "Lower object detection false alarm" → should be "lower false alarm rate."
  - "Miss rate" should specify the decrease in false negatives.
  - "Satisfies the requirements" → what requirements? Who sets them?
- **Introduction**
  - Lack of Clear Justification for SSD Selection – The introduction does not explain why SSD was chosen over YOLO or Faster R-CNN.
  - Missing Citations – Several references are missing or improperly formatted (e.g., "Error! Reference source not found.").
  - Give the abbreviations used at the beginning of the sentence.(ex, SSD, LAM, etc)
  - No explanation of YOLOv4-Tiny's weaknesses – Why was SSD chosen instead? Missing comparison with SSD – The introduction should mention how SSD differs from YOLO.
  - Unclear Explanation of Prior Work – The discussion of existing methods lacks coherence and should include a comparison table summarizing strengths and weaknesses.
  - Overly Technical Terms Introduced Too Early – Terms like CBAM, anchor boxes, feature pyramid appear without sufficient explanation.
- **Experiment and result analysis**
  - The "Experiment and Result Analysis" section is crucial for demonstrating the effectiveness of the proposed CBAM-SSD model. This section presents strong experimental validation but needs more details on dataset biases, training choices, computational cost, and real-world deployment feasibility.
  - Missing details on dataset composition – Where was the fire data collected? Where were these fire images taken? From real-world fires or simulations? Were there biases in the dataset?- No discussion on dataset bias – Are there regional, seasonal, or environmental biases?
  - Lack of justification for hyperparameters – Why was 300 epochs chosen? How was the learning rate tuned?
  - Image resizing details unclear – Why was 1000 pixels chosen as the threshold?
  - No mention of training time per epoch – How long did the full training process take?
  - No explanation of AP calculation – Did it use IoU thresholds (e.g., 0.5, 0.75, or averaged over multiple IoUs)?
  - Insufficient explanation of ablation studies – Needs more discussion on why CBAM outperforms SE-Net.
  - Computational efficiency not discussed – Does CBAM-SSD increase inference time compared to traditional SSD?
  - Comparison with real-world deployment – How would this model perform on edge devices (e.g., drones, mobile applications)?
- **Conclusion and discussion**
  - The "Conclusion and Discussion" section should provide a strong summary of findings, limitations, real-world implications, and future research directions. While the manuscript presents a well-structured conclusion, several areas require improvements.
  - The conclusion should be more impactful – It lacks a strong final statement on the model’s real-world benefits.
  - "Dual perception mechanism" is unclear – Does this refer to channel and spatial attention in CBAM?
  - No discussion on real-world deployment challenges – How does this model perform in edge devices, UAVs, or surveillance cameras?
  - Limitations are not fully addressed – What are the trade-offs of using CBAM-SSD?
  - Future work is too vague – The section should mention specific techniques for optimizing the model (e.g., quantization, federated learning).
  - Lacks a strong concluding remark – The last sentence should emphasize impact and real-world significance.
- **Reference**
  - Redundant or outdated references – Some citations refer to older versions of algorithms (e.g., YOLOv4 instead of YOLOv8).
