# Supplementary material for: Deep learning-based forest fire detection using an improved SSD algorithm with CBAM
Source: PLoS One. 2025 Nov 18;20(11):e0333574. doi: 10.1371/journal.pone.0333574 (PMC12626326; doi:10.1371/journal.pone.0333574)
Supplement: Attachment — Submitted filename: Response to Reviewers.docx [file pone.0333574.s005.docx]

Dear Editors and Reviewers:

Thank you for your letter and for the reviewers’ comments concerning our manuscript entitled “Research on forest fire detection based on improved SSD algorithm” (ID: PONE-D-24-53870). We greatly appreciate the valuable and constructive comments provided by the reviewers and editor, which have been instrumental in improving the quality and clarity of our manuscript. We have carefully addressed each point and revised the manuscript accordingly.

**Response to Narendra Khatri, Ph.D:**

1. We have revised the manuscript in accordance with the formatting requirements of PLOS ONE. We carefully reviewed all the comments provided by the reviewers and have made the corresponding revisions in the manuscript. In addition, we have corrected grammatical issues throughout the entire text.
2. In response to PLOS ONE request, we have also uploaded the source code to GitHub：https://github.com/zhangdiansheng1999/fire-detection.git
3. Our dataset was self-constructed and is owned by the Key Laboratory of RF Communication and Sensor Network in Jiangxi Province, China. The laboratory does not allow open distribution. Interested researchers may request access by contacting Professor Liu at: [ecjtu-tech@outlook.com](mailto:ecjtu-tech@outlook.com).

**Response to Reviewers:**

**Reviewer #1:**

***• Title***

*o Consider specifying "deep learning-based detection" to make the title more informative. Suggested title revision: "Deep Learning-Based Forest Fire Detection Using an Improved SSD Algorithm with CBAM"*

**The author’s answer:**

1) We have modified the title to: "Deep Learning-Based Forest Fire Detection Using an Improved SSD Algorithm with CBAM".

***• Abstract***

*o The phrase "high real-time and embeddability required for detection are achieved" is unclear and grammatically awkward.*

*o The term "random data enhancement" is vague—it should specify data augmentation techniques used.*

*o The purpose of "compensate for constraints in the data collection process" is unclear—what constraints?*

*o "Constraint information" is unclear—does it mean feature extraction constraints?*

*o "Channel (Channel) and space (Spatial)" is redundant—just say "channel and spatial features."*

*o The phrase "serial structure is utilized" is vague—explain why this structure was chosen.*

*o The phrase "traditional detection algorithm" is too vague—specify which algorithm (e.g., VGG-16-SSD).*

*o The term "average accuracy AP" is redundant—just say "AP (Average Precision)".*

*o The recall rate of 96.40%—is it for fire detection, smoke detection, or both?*

*o mAP (97.55%)—better to clarify that it is the mean average precision across all categories.*

*o "Lower object detection false alarm" → should be "lower false alarm rate."*

*o "Miss rate" should specify the decrease in false negatives.*

*o "Satisfies the requirements" → what requirements? Who sets them?*

**The author’s answer:**

1) Based on your suggestions and a summary of the manuscript, we have reorganized the abstract to highlight the challenges in forest fire detection, the adopted technical approach, experimental results, and contributions. The modifications in the abstract are marked in red in the revised version.
2) We removed the phrase "high real-time and embeddability required for detection are achieved" and further summarized the problem of forest fire detection: the varying scales and complex features of flame and smoke, as well as false positives and missed detections caused by environmental interference.
3) We have changed "random data enhancement" to data augmentation techniques and added the specific methods used.
4) We have revised "compensate for constraints in the data collection process" to: address problems such as insufficient and incomplete data during the collection process.
5) Regarding "Channel (Channel) and space (Spatial)", since we added the channel attention mechanism first and then the spatial attention mechanism to the SSD, we referred to it as a serial structure. This part has now been rewritten.
6) According to your suggestion, we changed "traditional detection algorithm" to baseline SSD.
7) Yes, we have changed "average accuracy AP" to AP50, and "mAP" to mAP@0.5. We have also clarified that the mAP@0.5 refers to the mAP@0.5 across all categories.
8) Since no unified standard has been set for forest fire detection, we revised the sentence to: provide an efficient, convenient, and accurate solution for forest fire detection.

***• Introduction***

*o Lack of Clear Justification for SSD Selection – The introduction does not explain why SSD was chosen over YOLO or Faster R-CNN.*

*o Missing Citations – Several references are missing or improperly formatted (e.g., "Error! Reference source not found.").*

*o Give the abbreviations used at the beginning of the sentence.(ex, SSD, LAM, etc)*

*o No explanation of YOLOv4-Tiny's weaknesses – Why was SSD chosen instead? Missing comparison with SSD – The introduction should mention how SSD differs from YOLO.*

*o Unclear Explanation of Prior Work – The discussion of existing methods lacks coherence and should include a comparison table summarizing strengths and weaknesses.*

*o Overly Technical Terms Introduced Too Early – Terms like CBAM, anchor boxes, feature pyramid appear without sufficient explanation.*

**The author’s answer:**

1) We used Word’s cross-reference function for citing references. When we modified the references, we failed to update the cross-referenced numbers in the main text, which led to such errors. We have corrected all reference formats in the manuscript. We apologize for our oversight.
2) According to your suggestion, we have added content in the introduction to explain YOLO, Faster R-CNN, and SSD, their respective advantages and disadvantages, and why we chose SSD. We have also added a paragraph introducing the structure of the paper.

***• Experiment and result analysis***

*o The "Experiment and Result Analysis" section is crucial for demonstrating the effectiveness of the proposed CBAM-SSD model. This section presents strong experimental validation but needs more details on dataset biases, training choices, computational cost, and real-world deployment feasibility.*

*o Missing details on dataset composition – Where was the fire data collected? Where were these fire images taken? From real-world fires or simulations? Were there biases in the dataset?- No discussion on dataset bias – Are there regional, seasonal, or environmental biases?*

*o Lack of justification for hyperparameters – Why was 300 epochs chosen? How was the learning rate tuned?*

*o Image resizing details unclear – Why was 1000 pixels chosen as the threshold?*

*o No mention of training time per epoch – How long did the full training process take?*

*o No explanation of AP calculation – Did it use IoU thresholds (e.g., 0.5, 0.75, or averaged over multiple IoUs)?*

*o Insufficient explanation of ablation studies – Needs more discussion on why CBAM outperforms SE-Net.*

*o Computational efficiency not discussed – Does CBAM-SSD increase inference time compared to traditional SSD?*

*o Comparison with real-world deployment – How would this model perform on edge devices (e.g., drones, mobile applications)?*

**The author’s answer:**

1) We have added Section 3.1 to explain the dataset in detail based on your comments. A figure showing the distribution of target numbers in the dataset has also been added.
2) We chose 300 epochs to ensure that the model converges sufficiently and achieves optimal performance. The learning rate was adjusted using cosine annealing, with a minimum value of 0.00002 and a maximum value of 0.002. We have described this in Section 3.2 of the revised manuscript.
3) Due to our oversight, we failed to mention the IoU threshold. In our experiments, the IoU threshold was always set to 0.5. This has now been clarified in Section 3.3.
4) We added a discussion in Section 3.4 explaining why CBAM outperforms SE-Net.
5) Compared with VGG-16-SSD, the inference time of our model remains nearly unchanged. As shown in Table 3, the FPS of VGG-16-SSD is 39.76, and that of our model is 39.42.
6) Since we have not yet deployed the model on UAVs, we have not conducted a comparison. Our next plan is to deploy the model on UAVs.

***• Conclusion and discussion***

*o The "Conclusion and Discussion" section should provide a strong summary of findings, limitations, real-world implications, and future research directions. While the manuscript presents a well-structured conclusion, several areas require improvements.*

*o The conclusion should be more impactful – It lacks a strong final statement on the model’s real-world benefits.*

*o "Dual perception mechanism" is unclear – Does this refer to channel and spatial attention in CBAM?*

*o No discussion on real-world deployment challenges – How does this model perform in edge devices, UAVs, or surveillance cameras?*

*o Limitations are not fully addressed – What are the trade-offs of using CBAM-SSD?*

*o Future work is too vague – The section should mention specific techniques for optimizing the model (e.g., quantization, federated learning).*

*o Lacks a strong concluding remark – The last sentence should emphasize impact and real-world significance.*

**The author’s answer:**

1) According to your suggestion, we have revised the conclusion.
2) The term "dual perception mechanism" refers to the addition of two CBAM modules in the SSD. As this expression may cause misunderstanding, we have changed "dual perception mechanism" to "mechanism".
3) We have not yet deployed the model on edge devices, UAVs, or surveillance cameras. Such deployment requires integration with device systems and construction of deployment platforms. We plan to implement this in the next stage of our work.
4) The unique advantages of CBAM-SSD in forest fire detection are discussed in Section 1.3. In addition, we summarized the disadvantages of CBAM-SSD in the conclusion: one limitation is that deployment on edge devices, UAVs, or surveillance cameras requires model lightweighting, as these devices lack sufficient computing power.

***• Reference***

*o Redundant or outdated references – Some citations refer to older versions of algorithms (e.g., YOLOv4 instead of YOLOv8).*

**The author’s answer:**

1) We have corrected the reference formats throughout the manuscript. We apologize for our oversight.

**Reviewer #2:**

1. *Writing Quality and Structure - The manuscript contains numerous grammatical, typographical, and formatting errors, such as:
   “Error! Reference source not found.” — appearing multiple times suggests broken citations from a word processor.
   Long and unclear sentences, e.g., "The mapping space and channel feature information can be aggregated..." lacks precision and clarity.
   Recommend to replace broken references and clean up figure labels and captions. Each figure should be referenced in the body text and explained.*

**The author’s answer:**

1) We have restructured the entire manuscript and improved grammatical correctness throughout.
2) We used the cross-reference function in Word for citations. When modifying the reference list, we failed to update the in-text reference numbers, resulting in errors like "Error! Reference source not found." We have now corrected all reference formats. We sincerely apologize for our carelessness.
3) According to the PLOS ONE submission guidelines, figure labels and captions should be retained in the manuscript. We followed these requirements. However, if this is considered inappropriate, we are willing to remove them.
2. *The paper reports only single-point values (e.g., mAP = 97.55%) without any standard deviation, variance, or confidence intervals. This makes it difficult to assess the statistical significance of the reported improvements, especially when the gain is marginal (e.g., a 1.53% improvement over baseline).
   There is no use of statistical tests (e.g., paired t-tests, Wilcoxon signed-rank test) to evaluate whether differences between models are statistically meaningful.
   Suggestion - Include standard deviation or confidence intervals for key metrics. Clearly state how many trials were run and whether the results are averaged. Apply statistical tests to support claims of superiority when differences are marginal.*

**The author’s answer:**

1) Thank you for your valuable suggestion. We also agree that including standard deviation or statistical testing is typically important for evaluating the significance of performance differences.
2) However, in our case, the reported AP50 values were obtained using a deterministic model on a fixed test set. We set fixed random seeds during training to avoid randomness, and disabled data augmentation during testing to ensure realism. Therefore, repeated evaluations produce identical results, and statistical testing is not applicable in this situation. We have added clarification regarding this in Section 3.3 of the revised manuscript.
3. *The dataset construction lacks detail—while the authors mention it was compiled using web crawlers and simulation, there is no discussion of ground truth annotation quality, data diversity, or whether any public datasets were included.*

**The author’s answer:**

1) We have added a dedicated section (Section 3.1) to explain our dataset source and data quality.
2) In addition, we included a figure showing the target distribution of the dataset to help illustrate its composition.

4. *The core techniques—CBAM, data augmentation, and SSD improvements—have been widely used and studied in various domains. The novelty lies primarily in their application to forest fire detection, but the paper does not explicitly justify why this combination is uniquely effective for that use case.
   Suggestions: Provide deeper insight into why CBAM specifically improves fire/smoke detection (e.g., are flame patterns especially sensitive to spatial context?).*

**The author’s answer:**

1) Thank you for your suggestion. In Section 1.3, we added three new paragraphs discussing the characteristics of fire detection, the limitations of SSD, and the advantages of CBAM.
5. *Consider benchmarking against CBAM + YOLOv5 or CBAM + RetinaNet to isolate the benefit of your integration with SSD versus other modern detectors.*

**The author’s answer:**

1) We added CBAM to YOLOv5, and the results are presented in Table 3. We also provided analysis of YOLOv5 with CBAM.
6. *Additionally, include a section highlighting novel insights or unique domain challenges to strengthen your contribution claim.*

**The author’s answer:**

1) According to your suggestion, we have revised the conclusion and added a discussion of novel insights and domain-specific challenges.

We deeply appreciate the reviewers’ and editor’s insightful comments and suggestions, which have been instrumental in refining our work. We hope the revised manuscript will be considered suitable for publication in PLOS ONE.

Sincerely,

Yueyuan Zhang

School of Information and Software Engineering,

East China Jiaotong University, Nanchang, China.

Email: zyyaney1981@hotmail.com
