# Supplementary material for: Deep learning-based forest fire detection using an improved SSD algorithm with CBAM
Source: PLoS One. 2025 Nov 18;20(11):e0333574. doi: 10.1371/journal.pone.0333574 (PMC12626326; doi:10.1371/journal.pone.0333574)
Supplement: Attachment — Submitted filename: PONE-D-24-53870_R1_ReviewRound2.docx [file pone.0333574.s004.docx]

**Deep learning-based forest fire detection using an improved SSD algorithm with CBAM**

The authors have made significant revisions based on the first round of review, which have substantially strengthened the manuscript.

- The authors have addressed the suggestion, and the revised title is clear and descriptive.
- The authors have made appropriate changes based on the suggestions. The abstract is now clearer, with terms more specifically defined and phrasing made more precise.
- The authors have satisfactorily addressed some points on first round, improving the clarity and structure of the introduction.
- The authors have made good progress in addressing first round concerns on Experiment and Result Analysis. They provided additional details on dataset construction and training, though further analysis of deployment feasibility (e.g., edge devices) might be useful in future versions.
- The conclusion is now more impactful, and the authors have clearly addressed the feedback regarding the "dual perception mechanism" and real-world deployment challenges. The mention of UAV deployment plans is a good addition.
- The authors have appropriately addressed the issues with citations.

Overall, the revisions have significantly strengthened the manuscript, and it appears ready for publication with minor adjustments related to :

- Several abbreviations are introduced without being defined or explained when first mentioned in the manuscript. Generally, it's best to avoid using abbreviations in the abstract of a paper, especially if the audience may not be familiar with them. However, if you must use abbreviations, spell out the full term followed by the abbreviation in parentheses the first time it's used, and then you can use the abbreviation throughout the rest of the abstract and the paper.
- The authors should explicitly state the calculation methods for performance metrics, especially mAP, and provide more context on how they chose thresholds like IoU (0.5) or AP50 (from reference?). A brief explanation of the metric calculation process would help the reader understand the significance of the results.
- Although the authors mention some limitations in the conclusion, the discussion could benefit from more depth, particularly in terms of trade-offs associated with using CBAM-SSD (Trade-offs between accuracy and computational efficiency? Potential overfitting?)
- Real-world application and deployment. It would be beneficial to see further exploration of real-world deployment, particularly regarding the integration of the model into UAVs and edge devices, as mentioned in the conclusion.
