# Supplementary material for: Deep learning-based forest fire detection using an improved SSD algorithm with CBAM
Source: PLoS One. 2025 Nov 18;20(11):e0333574. doi: 10.1371/journal.pone.0333574 (PMC12626326; doi:10.1371/journal.pone.0333574)
Supplement: Attachment — Submitted filename: Response_to_Reviewers_auresp_2.docx [file pone.0333574.s006.docx]

Dear Editors and Reviewers:

Thank you for your letter and for the reviewers’ comments concerning our manuscript entitled “Deep learning-based forest fire detection using an improved SSD algorithm with CBAM” (ID: PONE-D-24-53870). We greatly appreciate the valuable and constructive comments provided by the reviewers and editor, which have been instrumental in improving the quality and clarity of our manuscript. We have carefully addressed each point and revised the manuscript accordingly.

**Response to Narendra Khatri, Ph.D:**

1. The reference list cited in this paper does not include any retracted papers.
2. We have identified a spelling error in the evaluation criteria in Table 2. The correction has been made and highlighted in red in the "Revised Manuscript with Track Changes" file.
3. We have re-stated the financial disclosure in the Cover Letter.
4. We sincerely appreciate your guidance and your patience in providing assistance.

**Response to Reviewers:**

**Reviewer #1:**

Question1：

*• Several abbreviations are introduced without being defined or explained when first mentioned in the manuscript. Generally, it's best to avoid using abbreviations in the abstract of a paper, especially if the audience may not be familiar with them. However, if you must use abbreviations, spell out the full term followed by the abbreviation in parentheses the first time it's used, and then you can use the abbreviation throughout the rest of the abstract and the paper.*

**The author’s answer:**

We sincerely apologize for not identifying this issue during multiple rounds of review. We have now spelled out the full terms for abbreviations such as Faster R-CNN and SPPNet the first time they appear.

Question2：

*• The authors should explicitly state the calculation methods for performance metrics, especially mAP, and provide more context on how they chose thresholds like IoU (0.5) or AP50 (from reference?). A brief explanation of the metric calculation process would help the reader understand the significance of the results.*

**The author’s answer:**

In Section 3.3, we provide a detailed explanation of the calculation methods for Precision, Recall, AP, and mAP, and we also explain why we use 0.5 as the threshold for IoU.

Question3：

*• Although the authors mention some limitations in the conclusion, the discussion could benefit from more depth, particularly in terms of trade-offs associated with using CBAM-SSD (Trade-offs between accuracy and computational efficiency? Potential overfitting?)*

*• Real-world application and deployment. It would be beneficial to see further exploration of real-world deployment, particularly regarding the integration of the model into UAVs and edge devices, as mentioned in the conclusion.*

**The author’s answer:**

We have divided the conclusion into three parts: the achievements of CBAM-SSD, the limitations of CBAM-SSD, and the future directions of CBAM-SSD’s application. In the second part, we focus on discussing how to balance precision and computational efficiency when using CBAM-SSD.

We deeply appreciate the reviewers’ and editor’s insightful comments and suggestions, which have been instrumental in refining our work. We hope the revised manuscript will be considered suitable for publication in PLOS ONE.

Sincerely,

Yueyuan Zhang

School of Information and Software Engineering,

East China Jiaotong University, Nanchang, China.

Email: zyyaney1981@hotmail.com
